# Association between Mitral Valve Pathology and Ventricular Ectopy in the Pediatric Marfan Population

**DOI:** 10.1007/s00246-025-03963-w

**Published:** 2025-08-29

**Authors:** Saneeha Shahid, Peter F. Aziz, Iqbal El Assaad, Brendan J. Burke, Kenneth Zahka, Tara Karamlou, Justin T. Tretter, Maryann Patterson, Akash Patel

**Affiliations:** 1 Department of Pediatric Cardiology, Cleveland Clinic Children’s, 9500 Euclid Avenue, Cleveland, OH 44195 USA; 2 Department of Pediatric Cardiothoracic Surgery, Cleveland Clinic Children’s, 9500 Euclid Avenue, Cleveland, OH 44195 USA

**Keywords:** Marfan, Ventricular ectopy, Mitral annular disjunction, Mitral valve prolapse

## Abstract

Adult studies establish an association between mitral valve pathology, namely mitral annular disjunction (MAD) and mitral valve prolapse (MVP), and ventricular arrhythmias. Data in the pediatric Marfan population is limited. To assess the association between (1) MAD and ventricular ectopy (VE), non-sustained ventricular tachycardia (NSVT) and ventricular tachycardia (VT); (2) MVP and VE, NSVT and VT and (3) MAD and MVP in the pediatric Marfan population. We carried out a retrospective single center study from January 2001 to January 2022 including all patients with Marfan syndrome who were ≤ 21 years of age and had a cardiac rhythm monitor. Of the 32 patients included, 12 (38%) were female and 21 (66%) had a positive Fibrillin 1 variant. The mean age at echocardiogram was 13.5 ± 4.5 years and median duration of cardiac monitoring was 58 (32.5–190.5) hours. Sixteen (50%) had complex VE (couplets, triplets, and/or NSVT). Fourteen (44%) had couplets with median episodes per monitor of 2 (1–4), 1 (3%) being polymorphic and 6 (19%) with fast RR (R-R interval < 350 ms). Six (19%) had triplets with median episodes per monitor of 1 (1–1) and fast RR in 4 (13%). Four (13%) had NSVT. There is a high prevalence of complex VE in the pediatric Marfan population. MAD and MVP were not associated with complex VE however, all patients with triplets and NSVT had MVP, mostly bileaflet. MAD is positively associated with bileaflet MVP and bileaflet MVP is associated with more ventricular ectopy.

## Introduction

Even though aortic dilatation, mitral valve pathology and cardiomyopathy are the typical cardiac manifestations in Marfan syndrome, ventricular arrhythmia in the absence of cardiomyopathy has emerged as an important feature of this disease and is linked with both morbidity and mortality [1]. Many adult studies describe the ‘arrhythmic mitral valve syndrome’ and establish an association between mitral valve pathology, namely mitral annular disjunction (MAD) and mitral valve prolapse (MVP), and ventricular arrhythmias [2–4].

MAD, first described in the 1800s, is the atrial displacement of the posterior mitral valve leaflet hinge point within the AV junction [5]. Mitral valve pathology is hypothesized to cause abnormal leaflet traction and regional myocardial stretch leading to fibrosis and ectopy foci [3]. This contrasts with ventricular ectopy from left ventricular dilation and dysfunction from cardiomyopathy or meaningful mitral regurgitation.

There is scarce and conflicting data about mitral valve pathology and ventricular arrhythmias in the pediatric population. The Pediatric Heart Network (PHN) Marfan Trial showed a 7% prevalence of ventricular ectopy in the Marfan cohort with no association with MVP or mortality [6]. However, small cohorts and case reports have reported fatal ventricular arrhythmias in pediatric Marfan patients with MAD and MVP [2, 7].

Our aims were to assess the association between (1) MAD and ventricular ectopy (VE), non-sustained ventricular tachycardia (NSVT) and ventricular tachycardia (VT), (2) MVP and VE, NSVT and VT and (3) MVP and MAD in the pediatric Marfan population.

## Methods

This was a single center retrospective study. Chart review was completed for all patients with a diagnosis of Marfan syndrome (based on Ghent criteria and/or positive pathogenic Fibrillin 1 variant) who were ≤ 21 years of age and had a cardiac rhythm monitor between January 2001 and January 2022. Patients with incomplete data and lack of echocardiogram within 6 months of the cardiac monitor or prior mitral valve surgery were excluded from the study.

**Fig. 1:**
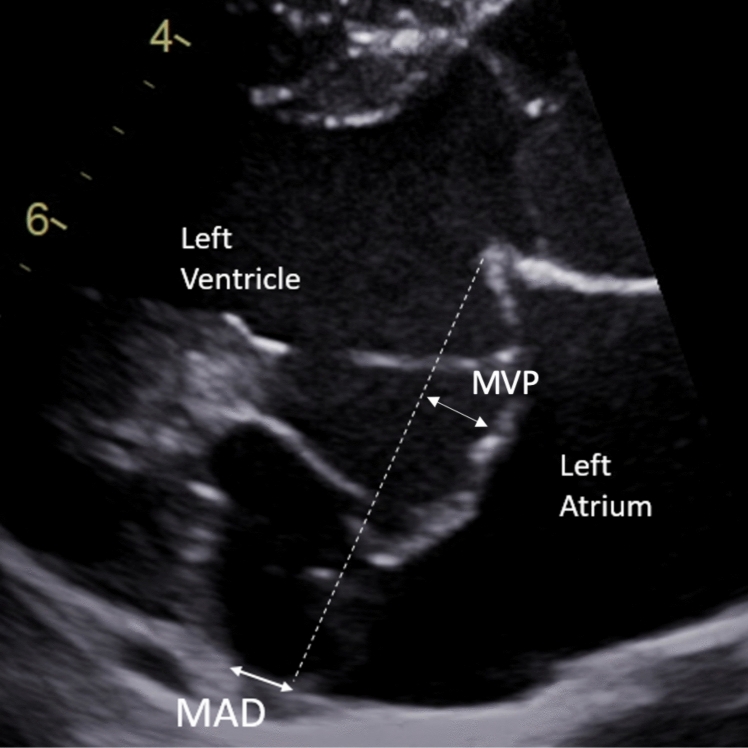
Parasternal long axis echocardiographic image showing atrial displacement of the posterior mitral valve leaflet hinge point within the AV junction (mitral annular disjunction) and mitral valve prolapse

Baseline demographics, clinical characteristics, cardiac monitor tracings and echocardiographic images were reviewed. MAD was assessed in the parasternal long axis on echocardiogram adjacent to the inferolateral left ventricular wall and atrial displacement ≥ 2 mm of the posterior mitral valve leaflet hinge point within the AV junction was considered to be a positive finding (Fig. 1) [5, 8]. A single observer measured the MAD to minimize inter-observer variability. MVP was defined as the bulging of mitral valve leaflets into the left atrium beyond the mitral annulus ≥ 2 mm during systole (Fig. 1) [9]. Complex ventricular ectopy was defined as couplets, triplets, NSVT or VT.

For statistical analysis, Shapiro–Wilk test was used to assess normality of all continuous variables in the study. Means ± standard deviation and t-test/ANOVA were used for continuous variables that were normally distributed and median (IQR) and rank-sum/Kruskal–wallis were used for continuous variables that were not normally distributed. Chi Square/Fisher’s test was used for analyses of categorical variables. *p* value of < 0.05 was considered significant.

This study was conducted in full accordance with all applicable Cleveland Clinic Research Policies and Procedures and all applicable federal and state laws and regulations including 45 CFR 46, and the HIPAA Privacy Rule. Cleveland Clinic Institutional Review Board approval was obtained.

## Results

### Baseline Characteristics

A total of 32 patients met inclusion criteria. Twelve (38%) were female and 21 (67%) had a pathogenic Fibrillin 1 variant. The mean age at echocardiogram was 13.5 ± 4.5 years and the median duration of cardiac monitoring was 58 (32.5–190.5) hours. Beta-blocker use for any reason was documented in 18 (56%) of patients which was determined based on the discretion of the primary cardiologist for mostly aortic root dilation. Only 3 (9%) reported ectopy related symptoms. 8 (25%) had MAD and 19 (59%) had MVP. The median MAD distance was 7.1 mm (Range 2.1–8.9 mm). There were no differences in baseline demographics between the groups with and without MAD and between the groups with and without MVP (Table 1).

**Table 1:** Baseline Demographics

Demographic Variables	Total	MAD	No MAD	p value	MVP	No MVP	p value
Number of patients	32	8 (25)	24 (75)		19 (59)	13 (41)	
Female	12 (38)	3 (38)	9 (38)	1	8 (42)	4 (31)	0.515
Age at Echo (years)	13.5 ± 4.5	11.6 ± 4.3	14.2 ± 4.4	0.16	13.8 ± 4.5	13.1 ± 4.6	0.67
Positive Fibrillin 1 mutation	21 (67)	5 (63)	16 (67)	0.83	11 (58)	10 (77)	0.233
Duration of cardiac monitoring (hours)	58.0 (32.5–190.5)	71.0 (31.0–212.5)	58.0 (35.5–190.5)	0.378	72.0 (47.0–329.0)	46.0 (25.5–69.0)	0.283
Beta Blocker use	18 (56)	7 (88)	11 (46)	0.053	12 (63)	6 (46)	0.473
Symptoms	3 (9)	1 (13)	2 (8)	1	1 (5)	2 (15)	0.535

Data presented as count (percent of total), mean ± standard deviation (SD), or median with interquartile range (IQR)

## Echocardiographic Findings

For echocardiographic variables (Table 2), 18 (56%) patients had mild or greater mitral regurgitation and 2 (6%) had mild aortic regurgitation with normal left ventricular end diastolic dimension (LVEDd) z scores in both instances. In the MAD group, the LVEDd z score was higher at 2.1 ± 1.9 compared to 0.56 ± 1.6 in the no MAD group (p value 0.037). Similarly, the LVEDd z score was higher in the MVP group at 1.4 ± 1.7 compared to 0.05 ± 1.4 in the no MVP group (*p* value 0.03). However, there were no differences in degree of mitral regurgitation or ejection fraction between the two groups.

**Table 2:** Echocardiographic Findings

Echo	Total	MAD	No MAD	p value	MVP	No MVP	p value
Number of patients	32	8	24		19	13	
Mitral regurgitation				0.552			0.13
No/trivial	14 (44)	2 (25)	12 (50)		6 (32)	8 (62)	
Mild	14 (44)	5 (63)	9 (38)		9 (47)	5 (39)	
Moderate	4 (13)	1 (13)	3 (13)		4 (21)	0 (0)	
Severe	0 (0)	0 (0)	0 (0)		0 (0)	0 (0)	
≥ Mild	18 (56)	6 (75)	12 (50)	0.412	13 (41)	5 (63)	0.149
LVEDd, z score	0.92 ± 1.7	2.1 ± 1.9	0.56 ± 1.6	0.037	1.4 ± 1.7	0.05 ± 1.4	0.03
LV EF	61.2 ± 5.7	62.7 ± 2.7	60.8 ± 6.3	0.487	61.8 ± 4.4	60.2 ± 7.4	0.462

LV EF, left ventricular ejection fraction; LVEDd, left ventricular end diastolic dimension. Data presented as count (percent of total), mean ± standard deviation (SD)

## Mitral Valve Pathology and Ventricular Ectopy

Twenty-seven (84%) had any ventricular ectopy with median ectopy per hour of 0.2 (0–1), 26 (81%) had single premature ventricular contractions (PVCs), 1(3%) had short-coupled PVC (R-R interval < 350 ms) and there was no R on T phenomenon (Table 3). There was no difference in presence or frequency of ventricular ectopy between groups with and without MVP or MAD.

**Table 3:** Characteristics of Ventricular Ectopy (VE)

Characteristics of VE	Total	MAD	No MAD	p value	MVP	No MVP	p value
Number of patients	32	8	24		19	13	
Presence of any ventricular ectopy	27 (84)	8 (100)	20 (83)	0.296	17 (90)	10 (77)	0.374
Ventricular Ectopy/Hr	0.2 (0–1)	0.35 (0.05–4.85)	0.2 (0–1)	0.821	0.15 (0 – 0.6)	0.3 (0.1 – 2.5)	0.337
Single PVCs	26 (81)	1 (8)	18 (75)	0.296	16 (84)	10 (77)	0.666
Multiform (≥ 3 morphologies)	4 (13)	2 (25)	2 (8)	0.548	2 (11)	2 (15)	0.602
R on T	0(0)	0 (0)	0 (0)		0 (0)	0(0)	
Short coupled PVC	1 (3)	0 (0)	1 (4)	1	0 (0)	1 (8)	0.36
Bigeminy	9 (28)	2 (25)	7 (29)	1	5 (26)	4 (31)	1
Trigeminy	1 (3)	0 (0)	1 (4)		0 (0)	1 (8)	0.406
Presence of complex ventricular ectopy	16 (50)	6 (75)	10 (42)	0.22	12 (63)	4 (31)	0.473
Couplets	14 (44)	5 (63)	9 (38)	0.252	10 (53)	4 (31)	0.289
No. of episodes/monitor	2.0 (1.0–4.0)	2.5 (1.0–5.0)	2.0 (1.5–4.0)		2.5 (1.0–4.0)	2.0 (-)	
Polymorphic (≥ 2 morphologies)	1 (3)	0 (0)	1 (4)		1 (5)	0 (0)	
Fast RR (< 350 ms)	6 (19)	3 (38)	3 (13)		4 (21)	2 (15)	
Triplets	6 (19)	2 (25)	4 (17)	0.625	6 (32)	0 (0)	0.059
No. of episodes/monitor	1 (1–1)	1 (1–1)	1 (1–1)		1 (1–1)	1 (1–1)	
Fast RR (< 350 ms)	4 (13)	1 (13)	3 (13)		4 (21)	0 (0)	
NSVT	4 (13)	2 (25)	2 (8)	0.254	4 (21)	0 (0)	0.128
Maximum HR > 180 bpm	2 (6)	1 (13)	1 (4)		2 (11)	0 (0)	

PVC, premature ventricular contraction; Data presented as count (percent of total), mean ± standard deviation (SD), or median with interquartile range (IQR)

Sixteen (50%) had complex ventricular ectopy (couplets, triplets and/or NSVT) (Table 3). Fourteen (44%) had couplets, 6 (19%) had triplets and 4 (13%) had NSVT. For the groups with and without MVP, even though there was no statistically significant difference for complex ventricular ectopy, there was a trend towards significance for triplets (p value 0.059). Of note, all patients with triplets and NSVT were in the MVP group even though this was not statistically significant. On more detailed analysis of location of MVP and number of leaflets involved (Table 4), there was a significant difference between no MVP, single MVP and bileaflet MVP with regards to the presence of ventricular ectopy and a trend towards significance with regards to triplets. For the groups with and without MAD, there was no statistically significant difference for complex ventricular ectopy.

**Table 4:** Characteristics of Ventricular Ectopy Based on Type of MVP

Characteristics of VE	Single leaflet MVP	Bileaflet MVP	No MVP	p value
Number of patients	5	14	13	
Presence of any ventricular ectopy	3 (60)	14 (100)	10 (77)	0.049
Presence of complex ventricular ectopy	2 (40)	10 (71)	4 (31)	0.504
Couplets	2 (40)	8 (57)	4 (31)	0.4
Triplets	2 (40)	4 (29)	0 (0)	0.051
NSVT	1 (20)	3 (21)	0 (0)	0.253

Data presented as count (percent of total)

Seven of 8 (88%) patients with MAD had bileaflet prolapse whereas only 7 of 24 (29%) patients without MAD had bileaflet prolapse (p value 0.01). Thus, bileaflet MVP was associated with MAD, but single leaflet valve disease did not have MAD. Based on the small number of patients with MAD and MVP on beta-blocker, the sample size was underpowered to provide definitive conclusions about beta-blocker effect on ventricular ectopy.

## Ventricular Arrhythmias in Marfan

**Fig. 2:**
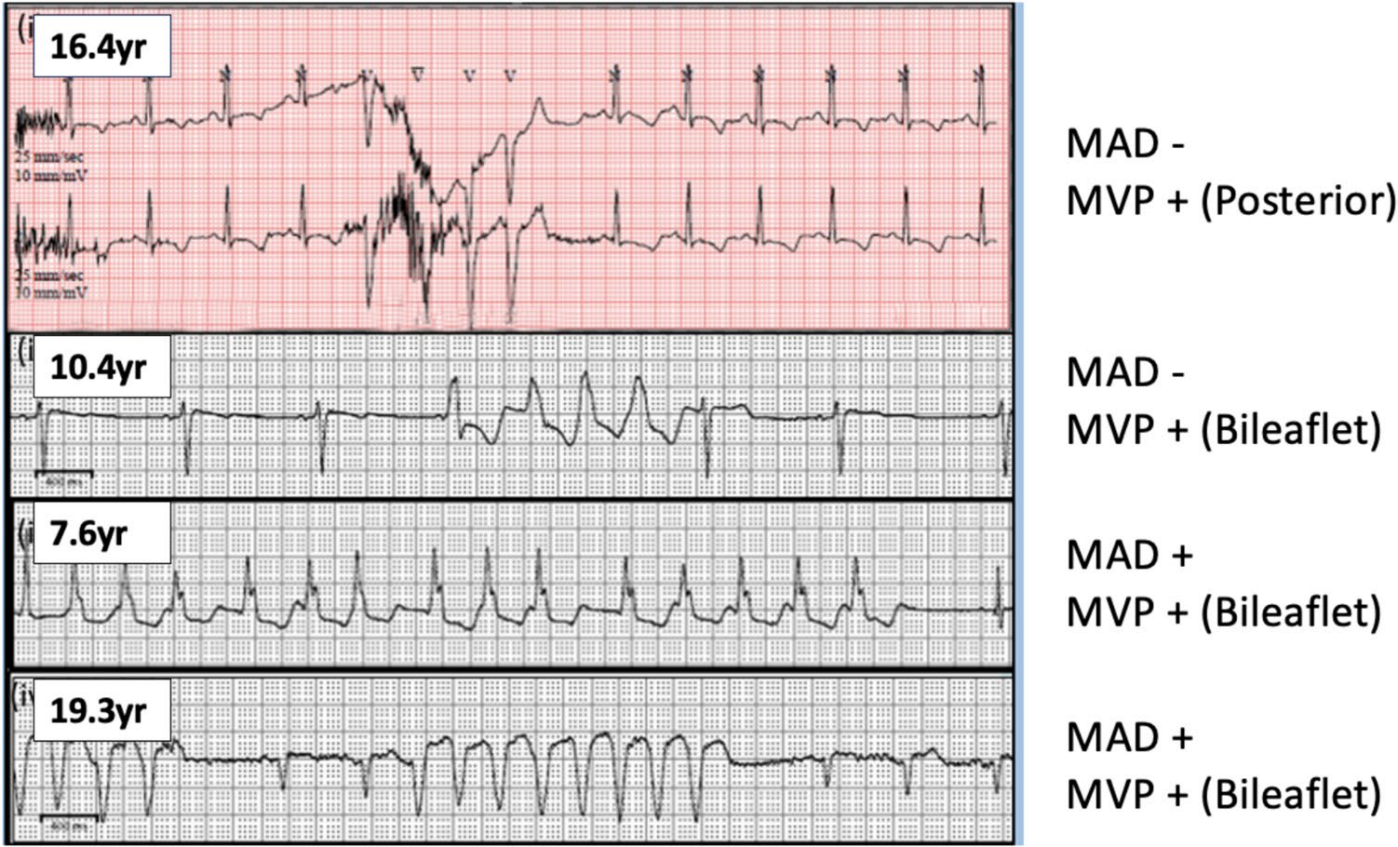
Cardiac monitor tracings of 5 episodes of non-sustained ventricular tachycardia among 4 patients. All 4 patients had mitral valve prolapse (MVP) (3 had bileaflet) and 2 patients also had mitral annular disjunction (MAD)

Four (13%) had NSVT with 2 (6%) having maximum heart rate of > 180 bpm during these episodes. There was a total of 5 NSVT episodes among 4 patients (Fig. 2). They were all monomorphic, the longest duration of NSVT was 6.8 s and the maximum heart rate was 222 bpm. These were all asymptomatic. All patients had normal LV size and function. Of note, all patients had MVP: 3 of them bileaflet and 1 posterior only even though this was not statistically significant. Two of these patients also had MAD. There was no sustained VT or sudden death in our population. Three of 4 were on beta-blockers (2 on atenolol for aortic dilation and 1 on carvedilol due to mild ventricular dysfunction and NSVT followed by metoprolol due to side effects).

## Discussion

Our study demonstrated a high prevalence of complex ventricular ectopy in the pediatric Marfan population. There was a positive association between MAD and bileaflet MVP, the latter of which is known to be associated with ventricular arrhythmias [3, 10, 11]. In our study as well, bileaflet MVP was associated with presence of ventricular ectopy. There was no other statistically significant association between mitral valve pathology and ventricular arrhythmias seen in this cohort.

We found a 50% prevalence of complex ventricular ectopy in our population with 13% having NSVT. This is a much higher prevalence than reported previously. The Pediatric Heart Network (PHN) Marfan trial found a 7% incidence of significant ventricular ectopy (defined as complex ventricular ectopy or > 10 PVCs/hour) [6]. Our findings suggest that a longer duration of cardiac monitoring, 58 (32.5–190.5) hours compared to 24 h in the PHN study, may detect more complex ectopy in this population. This suggests periodic monitoring may be beneficial in this group and contrasts with current adult recommendations which do not emphasize regular cardiac monitors in the asymptomatic Marfan population [12, 13]. However, in adult patients with MVP regardless of Marfan syndrome diagnosis or presence of symptoms, the 2022 European Heart Rhythm Association’s (EHRA) consensus statement recommends periodic Holter monitoring [14]. Our findings align with these recommendations especially considering all our patients with NSVT were asymptomatic. Improved arrhythmia detection in this population will enable better-informed decisions regarding future monitoring and treatment approaches.

The prevalence of MVP in young adults with sudden cardiac death (SCD) is reported as high as 7% [15]. Even though most patients with ventricular arrhythmias have associated meaningful mitral regurgitation, there have been multiple reports of SCD in patients with mitral valve disease without meaningful mitral regurgitation [10]. In our study, MVP was not associated with complex ventricular arrhythmias similar to the PHN Marfan trial [6]. However, all patients with triplets and NSVT had MVP and 3 of the 4 patients had bileaflet MVP. Even though this was not a statistically significant difference in our study, future studies with larger sample populations may elucidate this association better. Moreover, bileaflet mitral valve prolapse was associated with more ventricular ectopy in our study. MVP location and leaflet involvement was not separately assessed in the PHN trial. Bileaflet MVP has been positively associated with complex ventricular arrhythmias and sudden death in adult non-connective tissue disease populations in small cohorts [11, 15] although this has not been corroborated in larger studies [16]. The 2022 EHRA consensus statement also notes bileaflet MVP as an additional risk factor when considering arrhythmia risk.

Demolder et al. [2] reported a higher incidence of ventricular ectopy (42% vs 21%) and NSVT (39% vs 17%) in Marfan patients with MAD compared to those without MAD. The median age in their cohort was 25 years (range 2–64 years). They also noted that arrhythmic events (including 2 in the pediatric age group) occurred exclusively in the group with MAD with longer distances. Other adult studies have also identified MAD as a risk factor for ventricular arrhythmias independent of MVP [17]. In our study however, MAD was not associated with ventricular arrhythmias. This may be due to a combination of reasons. Since most studies are comprised of adult patients, this may indicate an accumulating risk with age. In addition, the rarity of arrhythmic events may require larger cohorts to detect statistical significance and additional risk factors.

It is important to note that cross-sectional imaging studies have shown that MAD is a common finding in the normal population with prevalence as high as 76–96% [18, 19]. In fact, the average length of disjunction was 3 mm in a normal adult population, with an upper range of 7 mm, involving approximately 39% of the posterior leaflet attachment.^16^ However, disjunction at the inferolateral ventricular wall is rare – reported as 5% among 2607 patients in the UK magnetic resonance imaging Biobank study by Zugwitz et al. [19]. Thus, despite being ubiquitous in the general population, MAD may indicate pathology when present in certain locations. The infero-lateral ventricular wall is the location assessed by echocardiogram in the parasternal long axis on echocardiogram. In the absence of cross-sectional imaging for every patient with Marfan syndrome, echocardiogram can thus be a useful modality to assess MAD. Furthermore, it may not simply be the length of disjunction involved, but also its circumferential extent, which portends pathophysiology. We also found a positive association between MAD and bileaflet MVP which is consistent with existent literature [20].

Even though none of our patients had documented cardiomyopathy, the LVEDd z score of patients with MAD and MVP was higher. The reason for this in unclear since there was no LV dysfunction or difference in degree of mitral or aortic regurgitation. This may suggest an underlying cardiomyopathy.

Our study is limited by its retrospective design and small sample size. The study may have been statistically underpowered to reveal an association between MVP and NSVT. The small sample size also increases the risk of type II error. In addition, presence of MAD could be under reported based on lack of evaluation with cross sectional imaging. This was not available for every patient so not included. MAD distance was not used as a quantitative variable due to the small sample size but has been reported as a risk factor for arrhythmias in the literature [21]. The majority of MVP was bileaflet and so analysis between anterior vs posterior leaflet prolapse was limited. It is our developing practice to routinely do cardiac monitors for all patients regardless of symptoms but longer duration of monitoring may have been done for symptomatic patients introducing some selection bias. Our study was also underpowered to evaluate the effect of beta-blocker use on ventricular ectopy in this population.

## Conclusions

There is a high prevalence of complex ventricular ectopy in the pediatric Marfan population which is mostly asymptomatic. Bileaflet MVP was associated with presence of ventricular ectopy in our population, but neither MAD nor MVP were associated with complex ventricular ectopy. MAD was also associated with bileaflet MVP and all patients with triplets and NSVT had MVP, mostly bileaflet suggesting a sub-group that warrants further delineation.

## Data Availability

The data that support the findings of this study are not openly available due to reasons of sensitivity and are available from the corresponding author upon reasonable request.
